# 2129 Let’s talk about sex: Does language create a barrier to women reporting and receiving treatment for dyspareunia in the Spanish-speaking community?

**DOI:** 10.1017/cts.2018.256

**Published:** 2018-11-21

**Authors:** Natalie Eisenach, Kimberly Swan, Dani Zoorob

**Affiliations:** University of Kansas Frontiers

## Abstract

OBJECTIVES/SPECIFIC AIMS: Dyspareunia is a type of female sexual dysfunction estimated to affect 8%–22% of women of all ages. There is concern that these statistics do not depict the true prevalence, because it frequently goes undiagnosed and untreated. By 2050, Latinos will make up 30% of the total population in the United States. As our patient population becomes more diverse, we need to ensure that our healthcare practices accommodate the changes. Our goals are to determine the prevalence of dyspareunia within our patient population and to identify if language impacts patients reporting symptoms of sexual dysfunction to their healthcare provider. METHODS/STUDY POPULATION: Our study is a convenience sample, cross-sectional survey of English and Spanish-speaking women, ages 18–45, who present to university-affiliated clinics. In total, 100 women from each language group will be studied. The survey will be completed in REDCap and will include the validated questionnaires for the Female Sexual Function Index (FSFI), Visual Analog Scales for pain, and Patient Global Impression of Severity and Improvement. Additional data on demographics and patient discussion of pain with their healthcare provider will be collected. RESULTS/ANTICIPATED RESULTS: The demographic and pain discussion questions will identify reporting rates. The FSFI score will be used to identify patients with sexual dysfunction and dyspareunia and calculate the prevalence in each language group. The domains will be analyzed to assess variations between populations. DISCUSSION/SIGNIFICANCE OF IMPACT: Dyspareunia has a great impact on patients’ quality of life when untreated. This study will allow us to identify barriers to diagnosing and treating cases of dyspareunia. If we detect differences in reporting rates between the language groups, future research could be tailored and conducted to identify the specific problems in communication. With this knowledge, we can improve how we discuss sexual health in clinic and ultimately improve quality of care for all patients.

